# Drug discontinuation before contrast procedures and the effect on acute kidney injury and other clinical outcomes: a systematic review protocol

**DOI:** 10.1186/s13643-018-0701-1

**Published:** 2018-02-21

**Authors:** Swapnil Hiremath, Jeanne Françoise Kayibanda, Benjamin J. W. Chow, Dean Fergusson, Greg A. Knoll, Wael Shabana, Brianna Lahey, Olivia McBride, Alexandra Davis, Ayub Akbari

**Affiliations:** 1 The Ottawa Hospital and the University of Ottawa, 1967 Riverside Drive, Ottawa, ON K1H7W9 Canada; 2 Department of Global Health and Social Medicine, Harvard Medical School, Boston, MA 02115 USA; 3 University of Ottawa Heart Institute, 40 Ruskin St, Ottawa, ON K1Y 4W7 Canada; 4 Ottawa Hospital Research Institute, Centre for Practice Changing Research, 1053 Carling Ave, Ottawa, ON K1Y 4E9 Canada; 5 Kidney Research Centre, The Ottawa Hospital, 1967 Riverside Drive, Ottawa, ON K1H7W9 Canada; 6 The Ottawa Hospital, 1967 Riverside Drive, Ottawa, ON K1H7W9 Canada

**Keywords:** Contrast nephropathy, Contrast-induced acute kidney injury, Acute kidney injury, Metformin, Renin-angiotensin system blockade, Diuretic, Ace inhibitors, Angiotensin receptor blockers, Non-steroidal anti-inflammatory drugs, Contrast imaging

## Abstract

**Background:**

Contrast-induced acute kidney injury (CI-AKI) is defined as worsening of renal function after the administration of iodinated contrast material. In patients with cardiovascular disease, kidney disease, and/or diabetes, renin-angiotensin system blockers, non-steroidal anti-inflammatory drugs, diuretics, and metformin can increase the risk of CI-AKI when undergoing contrast imaging. Despite CI-AKI being the leading iatrogenic cause of acute kidney injury, there is a lack of sufficient scientific evidence supporting which drugs should be stopped, when they should be stopped, and when they should be resumed. The purpose of this systematic review is to assess (1) the effect of withholding medication before contrast procedures on the risk of CI-AKI and other clinical outcomes and (2) the incidence of adverse events occurring after withholding these drugs prior to contrast procedures. This protocol has been registered with PROSPERO, https://www.crd.york.ac.uk/PROSPERO/display_record.asp?ID=CRD42016033178.

**Methods:**

An information specialist will assist in searching MEDLINE, Embase, and the Cochrane Library databases to identify randomized controlled trials, observational studies, case reports, and case series. Relevant abstracts from professional society meetings and web-based registries of clinical trials will also be included. Studies included will compare patients aged ≥ 18 years instructed to continue taking the drugs of interest and those advised to stop taking them before undergoing contrast procedures. If these drugs are not withheld prior to contrast procedures, the studies must compare patients who are administered these drugs and those who are not before undergoing contrast procedures. Two reviewers will independently screen the titles and abstracts of the studies obtained from the search using pre-defined inclusion criteria and will then extract data from the full texts of selected studies. The quality of the studies will be assessed by two independent reviewers using the Cochrane Risk of Bias 2.0 tool for randomized trials and the Newcastle-Ottawa Scale for observational studies.

**Discussion:**

This systematic review will provide a synthesis of current evidence on the discontinuation of drugs prior to contrast procedures and its effect on CI-AKI and other clinical outcomes. These findings will provide clinicians with guidelines and serve as a strong research base for future studies in this field.

**Systematic review registration:**

PROSPERO CRD42016033178

**Electronic supplementary material:**

The online version of this article (10.1186/s13643-018-0701-1) contains supplementary material, which is available to authorized users.

## Background

Iodinated contrast material is commonly used in many diagnostic and therapeutic procedures, including cardiac catheterization and computed tomography scanning [1]. Contrast-induced acute kidney injury (CI-AKI) is defined as the worsening of renal function after the administration of iodinated contrast material [1–3]. It is the leading iatrogenic and thus potentially preventable cause of acute kidney injury (AKI) [4]. Most clinical guidelines recommend holding renin-angiotensin system (RAS) blockers (angiotensin-converting enzyme inhibitors [ACEI], angiotensin receptor blockers [ARB], and mineralocorticoid antagonists), non-steroidal anti-inflammatory drugs (NSAIDs), diuretic, and metformin in patients with diabetes, kidney diseases, and/or cardiovascular diseases prior to the administration of contrast material in order to reduce the risk of CI-AKI [3, 5, 6]. However, there is a lack of sufficient scientific evidence supporting which drugs should be stopped, when they should be stopped, and when they should be restarted after contrast procedures.

The effects of drug discontinuation have been reported in patients advised to stop certain drugs prior to surgery, dental treatments, and other interventional procedures [7]. Discontinuing drugs leads to the risk that they may not be resumed after the intervention and lead to withdrawal effects [8, 9]. For example, discontinuation of metformin may result in hyperglycemia and the need for the institution of insulin, as well as weight gain [9].

We have two overall objectives in this systematic review: firstly, what is the effect of holding RAS blockers, NSAIDs, diuretics or metformin before the administration of contrast media on the risk of CI-AKI and related renal complication in patients who undergo contrast procedures? Secondly, what are the frequencies of clinical adverse events occurring after withdrawal of RAS blockers, NSAIDs, diuretics, or metformin prior to the administration of contrast media in patients who undergo contrast procedures?

## Methods

### Aims

Specific objectives are to summarize available evidence on (1) the risk of CI-AKI and other clinical outcomes [including renal outcomes, such as the need for renal replacement therapy (RRT), prolonged hospitalization, lactic acidosis, and death] amongst patients who underwent contrast procedures in whom these drug classes (specifically RAS blockers, NSAIDs, diuretics, and metformin) were withheld compared to those in whom they were not and (2) the incidence of adverse effects occurring after withholding RAS blockers, NSAIDs, diuretics, or metformin prior to contrast administration amongst patients who underwent contrast procedures.

### Study design

This systematic review will be performed to assess the effect of medication restriction before the administration of contrast material on the risk of subsequent CI-AKI and other clinical outcomes. It will adhere to the Preferred Reporting Items for Systematic Reviews and Meta-Analyses (PRISMA) statement, using the Cochrane Risk of Bias (RoB) 2.0 tool for individually randomized, parallel group trials, and the Newcastle-Ottawa Scale (NOS) for observational studies for quality assessment [10–12]. This protocol is reported in accordance with the PRISMA-P 2015 checklist (see Additional file 1).

### Eligibility criteria

#### Types of studies

The review will include randomized controlled trials, observational studies, case reports, and case series.

#### Patient population

This review will consider all studies conducted amongst adult patients aged ≥ 18 years who underwent contrast procedures, including contrast-enhanced computed tomographies (CT scans), cardiac catheterizations, and other angiographic procedures. The patient population will also be one receiving at least one of the drug classes to be studied (i.e., RAS blockade, diuretics, NSAIDs, or metformin).

#### Intervention

The exposure to be assessed will be withholding of the drug prior to the contrast procedure. The drugs to be studied include RAS blockade (renin inhibitor and aliskerin; ACE-I: captopril, cilazapril, enalapril, fosinopril, lisinopril, perindopril, quinapril, ramipril, trandolapril, benazepril, and moexipril; ARBs: candesartan, valsartan, eprosartan, irbesartan, losartan, telmisartan, and olmesartan), NSAIDs (aspirin, ibuprofen, and naproxen), loop diuretics (furosemide, bumetanide, ethacrynic acid, and torsemide), thiazide diuretics (hydrochlorothiazide, chlorothiazide, methylclothiazide, and bendrofluazide), thiazide-like diuretics (metolazone, chlorthalidone, and indapamide), and metformin.

In studies where the drugs of interest are not withheld in patients prior to contrast procedures, a comparison will be made between patients who are administered the drugs of interest (RAS blockade, diuretics, NSAIDs, or metformin) before undergoing contrast procedures and patients who are not administered the drugs prior to contrast procedures, thus serving as controls.

### Information sources and search strategy

Electronic bibliographic databases, including MEDLINE, Embase, and the Cochrane Library will be systematically searched with no restrictions on language. The search will be conducted by an information specialist (AD) who has developed a comprehensive search strategy and follows the Peer Review of Electronic Search Strategies (PRESS) recommendations [13]. The search terms were adapted for the different databases using a combination of Medical Subject Heading (MeSH) and relevant keywords contained in titles and abstracts. Articles from 1946 to the present time, from 1947 to December 21 of 2016, and from November 2016 will be searched using MEDLINE, Embase, and the Cochrane Library, respectively. See Table 1 for details of the search strategy for MEDLINE.

**Table 1:** Search strategy for MEDLINE

MEDLINE search strategy
1 exp. Contrast Media/
2 (contrast media or contrast medium or contrast material$ or contrast agent$ or contrast procedure* or contrast dye or contrast exposure or contrast-based or contrast induced or radiographic contrast).tw,kw. (63554)
3 (radiocontrast or radio contrast).tw,kw.
4 exp. Triiodobenzoic Acids/
5 (ioxithalamate or meglumine or iopamidol or iohexol or iopromide or iotrolan or iodixanol or ioversol).tw,kw.
6 or/1–5
7 (nephritis or nephropath$ or nephrotoxic$).tw,kw.
8 ((impair$ or damag$ or reduc$ or dysfunction or injur$ or toxic$) adj2 (renal or kidney)).tw,kw.
9 exp. nephritis/ or diabetic nephropathies/
10 exp. renal insufficiency/ or ((renal or kidney) adj insufficiency).tw,kw.
11 Kidney/de [Drug Effects]
12 exp. Kidney Diseases/ci [Chemically Induced]
13 Hyperglycemia/ or hyperglyc?em*.tw,kw.
14 Acidosis, Lactic/ or lactic acidosis.tw,kw.
15 Pulmonary Edema/ or (pulmonary edema or lung edema).tw,kw.
16 ((cardiac or cardiovascular) adj2 (outcome* or event*)).tw,kw.
17 Myocardial Infarction/ or heart attack.tw,kw. (171162)
18 Stroke/ or Ischemic Attack, Transient/ or (stroke or transient isch?emic attack*).tw,kw.
19 Hypertension/ or hypertens*.tw,kw.
20 weight gain/ or (weight adj2 gain*).tw,kw.
21 (adverse adj2 (effect* or event*)).tw,kw.
22 or/7–21
23 6 and 22
24 (contrast-induced nephr$ or contrast-associated nephr$).tw,kw.
25 (Contrast-induced acute kidney injur$ or ci-aki or ciaki or caaki).tw,kw.
26 cin.tw,kw.
27 or/24–26
28 23 or 27
29 exp. Angiotensin-Converting Enzyme Inhibitors/
30 angiotensin converting enzyme inhibitor$.tw,kw.
31 ACE inhibitor$.tw,kw.
32 (Captopril or Cilazapril or Enalapril or Enalaprilat or Fosinopril or Lisinopril or Perindopril or Ramipril or Teprotide).tw,kw.
33 exp. Angiotensin Receptor Antagonists/
34 (angiotensin adj2 block$).tw,kw.
35 (AT1 block$ or AT 1 block$).tw,kw.
36 (RAAS block$ or renin-angiotensin-aldosterone block$).tw,kw.
37 (ACEI or ACEIs or ARB or ARBs).tw,kw.
38 exp. Anti-Inflammatory Agents, Non-Steroidal/
39 (non-steroidal anti-inflammator$ or nonsteroidal anti-inflammator$ or nsaid or nsaids).tw,kw.
40 (ibuprofen* or aceclofenac* or diclofenac* or fenbufen* or fenoprofen or flurbiprofen or indomethacin or ketoprofen or mefanamic acid or nabumetone or naproxen or phenylbutazone or piroxicam or sulindac or tolmetin or antipyrine or phenazone or tenoxicam* or aminopyrine or curcumin or clofazimine).tw,kw.
41 (dimethylguanylguanidine or dimethylbiguanidine or glucophage or diabex or formet or diaformix or glucohexal or glucomet or glucovance or metforbell or diaformin).tw,kw.
42 Biguanides/ or Metformin/ or (biguanide* or metformin).tw,kw.
43 exp. Diuretics/ or diuretic$.tw,kw.
44 (furosemide or bumetanide or torsemide or ethacrynic acid).tw,kw.
45 exp. mineralocorticoid receptor antagonists/
46 aldosterone antagonist$.tw,kw.
47 spironolactone.tw,kw. (5834)
48 Renin/ai [Antagonists & Inhibitors]
49 ((renin or renin-angiotensin) adj2 inhibit$).tw,kw.
50 aliskiren.tw,kw.
51 or/29–50
52 28 and 51
53 Withholding Treatment/
54 (withhold$ or with hold$ or withdraw$ or discontinu$).tw,kw.
55 53 or 54
56 6 and 51 and 55
57 52 or 56
58 animals/ not humans/
59 57 not 58

The literature search will be extended by (1) reviewing the bibliographic reference lists of studies selected from electronic databases and (2) identifying relevant abstracts from relevant professional society meetings (from the fields of nephrology and cardiology).

### Study records

#### Data management and selection process

Articles will be imported into Microsoft Excel, and duplicates will be removed. The most relevant article per data source/analysis will be retained. Two reviewers (BL and OM) will independently screen the titles and abstracts of each potential relevant article to determine whether they meet the inclusion criteria. If an abstract is absent, the full text will be reviewed. Articles that meet the inclusion criteria will have their full texts obtained. Each independent reviewer will then screen the full texts of potentially relevant articles. Any disagreements between the independent reviewers will be mediated by a third reviewer (SH). Reviewers will not be blinded to the authors or journals when screening articles.

#### Data collection process

A data extraction template will be created by the principal investigator (SH) in Microsoft Excel and modified by feedback from two independent reviewers (BL and OM) to ensure that complete data is obtained. Any disagreements between the two independent reviewers (BL and OM) will be mediated by a third reviewer (SH). Reviewers will not be blinded to the authors or journals during this process.

### Data items

Variables for which data will be sought in the full text or abstract, if no full-text is available, of each selected study of interest include the following: (1) title, (2) first author, (3) design, (4) country, (5) publication year, (6) funding body (industry or drug company), (7) route of contrast (arterial or venous), (8) type of contrast (low/iso-osmolar or the actual name), (9) inclusion criteria, (10) exclusion criteria, (11) primary endpoint, (12) secondary endpoint, (13) drug(s) of interest, and (14) group (intervention and control).

In addition, variables for which demographic data will be sought in each group consist of the following: (1) sample size, (2) sex (%: male/female), (3) mean age (years), (4) diabetes, (5) definition of chronic kidney disease (CKD) or renal insufficiency, (6) definition of contrast-induced nephropathy (CIN) or contrast-induced acute kidney injury (AKI), (7) definition of acute kidney injury (AKI) or acute renal failure (ARF), (8) mean contrast volume (ml), and (9) smoking status (%).

Furthermore, variables for which kidney function will be sought in each group include the following: (1) mean baseline serum creatinine level (mg/dl) and standard deviation, (2) mean baseline glomerular filtration rate (GFR) (ml/min/1.73 m^2^) and standard deviation, (3) mean post serum creatinine level (mg/dl) and standard deviation, (4) mean post glomerular filtration rate (GFR) (ml/min/1.73 m^2^) and standard deviation, (5) incidence of CKD or renal insufficiency (actual number), (6) incidence of CIN or CI-AKI (actual number), and (7) incidence of AKI or ARF (actual number).

Lastly, co-interventions and adverse events will serve as other variables for data extraction.

### Outcomes and prioritization

The primary outcome of interest will be the risk of CI-AKI occurring after withholding the drugs. CI-AKI is a sudden deterioration in renal function following the recent intravascular administration of a contrast medium in the absence of another nephrotoxic event. It will be defined as either of the following: (1) a relative increase in serum creatinine (SCr) > 25% or an absolute increase in SCr ≥ 0.5 mg/dl (44 μmol/L) over baseline assessed at 48 to 72 h after radiological procedure and (2) a percentage increase of SCr ≥ 50% or an increase in SCr by ≥ 0.3 mg/dl (26.5 μmol/L) at or within 48 h after contrast medium administration (Acute Kidney Injury Network) [14].

The secondary outcome of interest will be the frequency of adverse effects occurring after withholding the drugs. More specifically, the incidence of the following events will be reported: (1) drugs not being restarted and (2) rebound hypertension, hyperglycemia, weight gain, or pain.

Additional outcomes that will be studied include other renal outcomes, such as the need for RRT and persistent renal dysfunction, as well as other morbidity outcomes, such as prolonged hospitalization, risk of lactic acidosis, and mortality outcome.

### Risk of bias in individual studies

The study quality and the presence of potential bias within individual studies will be done at both the outcome level and the study level. Two reviewers (BL and OM) will complete the assessment independently. Randomized controlled trials will be evaluated using the Cochrane Risk of Bias (RoB) 2.0 tool for individually randomized, parallel group trials [11]. The Newcastle-Ottawa Scale (NOS) will be used to evaluate observational studies [12]. A table comprised of study characteristics will illustrate the results from this methodology quality assessment.

### Data synthesis

Study data will be quantitatively synthesized by first assessing heterogeneity to examine whether the estimates from included studies could be pooled. This will be done using the *χ*^2^ and the *I*^2^ statistics, which test the hypothesis of homogeneity and quantifies the percentage of total variation across the studies due to heterogeneity, respectively. An *I*^2^ value < 60% will be considered acceptable for pooling results into a meta-analysis. Risk ratios (RR) and their 95% confidence interval (CI) will be calculated from the data generated by each study that is included. The overall effects and their 95% CI will be obtained using a random-effects model as described by DerSimonian and Laird [15]. RCTs and observational studies will be pooled separately. We will follow the “Meta-analysis of Observational Studies in Epidemiology” (MOOSE) guidelines while performing quantitative synthesis and reporting of the observational studies [16]. All analysis will be performed using the Comprehensive Meta-analysis software (version 2.2.046, Biostat Inc., Englewood, NJ, USA). In cases where quantitative synthesis is not appropriate because it is not possible to conduct meta-analysis due to the heterogeneity of estimates reported by studies, a qualitative narrative synthesis of the evidence will be performed. This will summarize the key characteristics of the studies included (i.e., types of studies, setting, number of participants, methods of analysis, etc.), the methodological quality of studies included, estimates reported, as well as size and direction of effects.

### Meta-bias assessment

Additional analyses that will be performed include sensitivity and multivariate meta-regression analyses in order to assess the effects of some clinical factors and socio-demographic characteristics reported in included studies (i.e., baseline SCr, age, sex, and time/duration of drug withholding) on the meta-analyses estimates.

Visual assessment of the funnel plot and the Egger’s statistic will be used to assess for both the presence and statistical significance of publication bias across studies [17].

## Discussion

The purpose of this systematic review is to identify, collect, and summarize the current evidence on the discontinuation of drugs prior to contrast procedures and its effect on CI-AKI and other clinical outcomes, such as the need for RRT, prolonged hospitalization, lactic acidosis, and death. More specifically, this study will aim to compare patients in whom these drugs were withheld and those in whom they were continued, prior to the exposure of contrast material, in order to better assess the risk of CI-AKI and other clinically important outcomes. An analysis of the incidence of adverse effects, which include drugs not being restarted, rebound, hypertension, and hyperglycemia or weight gain, occurring following the event of withholding these drugs will also be done.

The information from this systematic review will help advice cardiologists, radiologists, nephrologists, and other clinicians of the current evidence for interventions to prevent CI-AKI. In addition, this synthesis will enable clinicians to have a better understanding about which drugs should be stopped, when they should be stopped, and when they should be restarted after exposure to contrast material. This will allow patients at risk of developing CI-AKI or other adverse clinical outcomes to receive the proper care and treatment. These findings will ultimately provide a robust base to update clinical guidelines with new evidence-based recommendations and establish a strong research base for future studies in this subject field.

## Additional file

Additional file 1: PRISMA-P checklist (DOCX 25 kb)

## Abbreviations

ACEI: Angiotensin converting enzyme inhibitor
AKI: Acute kidney injury
ARB: Angiotensin receptor blocker
ARF: Acute renal failure
CI: Confidence interval
CI-AKI: Contrast-induced acute kidney injury
CIN: Contrast-induced nephropathy
CKD: Chronic kidney disease
CT: Computed tomography
GFR: Glomerular filtration rate
NSAIDs: Non-steroidal anti-inflammatory drugs
RAS: Renin-angiotensin system
RR: Risk ratio
RRT: Renal replacement therapy
SCr: Serum creatinine
