# Supplementary material for: Drug discontinuation before contrast procedures and the effect on acute kidney injury and other clinical outcomes: a systematic review protocol
Source: Syst Rev. 2018 Feb 21;7:34. doi: 10.1186/s13643-018-0701-1 (PMC5822653; doi:10.1186/s13643-018-0701-1)
Supplement: Supplementary file 1 — PRISMA-P checklist (DOCX 25 kb) [file 13643_2018_701_MOESM1_ESM.docx]

**PRISMA-P 2015 Checklist**

| Section/topic | # | Checklist item | Information reported | | Line number(s) |
| --- | --- | --- | --- | --- | --- |
|  |  |  | **Yes** | **No** |  |
| ADMINISTRATIVE INFORMATION | | | | | |
| Title | | | | | |
| Identification | 1a | Identify the report as a protocol of a systematic review | x |  | 1-4 |
| Update | 1b | If the protocol is for an update of a previous systematic review, identify as such |  | x | NA |
| Registration | 2 | If registered, provide the name of the registry (e.g., PROSPERO) and registration number in the Abstract | x |  | 40-42 |
| Authors | | | | | |
| Contact | 3a | Provide name, institutional affiliation, and e-mail address of all protocol authors; provide physical mailing address of corresponding author | x |  | 6-26 |
| Contributions | 3b | Describe contributions of protocol authors and identify the guarantor of the review | x |  | 302-305 |
| Amendments | 4 | If the protocol represents an amendment of a previously completed or published protocol, identify as such and list changes; otherwise, state plan for documenting important protocol amendments | x |  | NA |
| Support | | | | | |
| Sources | 5a | Indicate sources of financial or other support for the review | x |  | 299-301 |
| Sponsor | 5b | Provide name for the review funder and/or sponsor | x |  | 299-301 |
| Role of sponsor/funder | 5c | Describe roles of funder(s), sponsor(s), and/or institution(s), if any, in developing the protocol | x |  | 299-301 |
| INTRODUCTION | | | | | |
| Rationale | 6 | Describe the rationale for the review in the context of what is already known | x |  | 68-88 |
| Objectives | 7 | Provide an explicit statement of the question(s) the review will address with reference to participants, interventions, comparators, and outcomes (PICO) | x |  | 91-97 |
| METHODS | | | | | |
| Eligibility criteria | 8 | Specify the study characteristics (e.g., PICO, study design, setting, time frame) and report characteristics (e.g., years considered, language, publication status) to be used as criteria for eligibility for the review | x |  | 117-143 |
| Information sources | 9 | Describe all intended information sources (e.g., electronic databases, contact with study authors, trial registers, or other grey literature sources) with planned dates of coverage | x |  | 145-159 |
| Search strategy | 10 | Present draft of search strategy to be used for at least one electronic database, including planned limits, such that it could be repeated | x |  | 312 |
| *STUDY RECORDS* | | | | | |
| Data management | 11a | Describe the mechanism(s) that will be used to manage records and data throughout the review | x |  | 162-165 |
| Selection process | 11b | State the process that will be used for selecting studies (e.g., two independent reviewers) through each phase of the review (i.e., screening, eligibility, and inclusion in meta-analysis) | x |  | 164-171 |
| Data collection process | 11c | Describe planned method of extracting data from reports (e.g., piloting forms, done independently, in duplicate), any processes for obtaining and confirming data from investigators | x |  | 172-178 |
| Data items | 12 | List and define all variables for which data will be sought (e.g., PICO items, funding sources), any pre-planned data assumptions and simplifications | x |  | 178 -202 |
| Outcomes and prioritization | 13 | List and define all outcomes for which data will be sought, including prioritization of main and additional outcomes, with rationale | x |  | 203-220 |
| Risk of bias in individual studies | 14 | Describe anticipated methods for assessing risk of bias of individual studies, including whether this will be done at the outcome or study level, or both; state how this information will be used in data synthesis | x |  | 221-229 |
| *DATA* | | | | | |
| Synthesis | 15a | Describe criteria under which study data will be quantitatively synthesized | x |  | 230-236 |
|  | 15b | If data are appropriate for quantitative synthesis, describe planned summary measures, methods of handling data, and methods of combining data from studies, including any planned exploration of consistency (e.g., *I* ^2^, Kendall’s tau) | x |  | 236-241 |
|  | 15c | Describe any proposed additional analyses (e.g., sensitivity or subgroup analyses, meta-regression) | x |  | 251-255 |
|  | 15d | If quantitative synthesis is not appropriate, describe the type of summary planned | x |  | 241-248 |
| Meta-bias(es) | 16 | Specify any planned assessment of meta-bias(es) (e.g., publication bias across studies, selective reporting within studies) | x |  | 256-258 |
| Confidence in cumulative evidence | 17 | Describe how the strength of the body of evidence will be assessed (e.g., GRADE) |  | x | NA |
